# (*E*)-*N*′-(2,4-Dichloro­benzyl­idene)-3-nitro­benzohydrazide

**DOI:** 10.1107/S1600536810049470

**Published:** 2010-11-30

**Authors:** Fu-Lin Mao, Chun-Hua Dai

**Affiliations:** a Jiangsu Provincial Key Laboratory of Coastal Wetland Bioresources and Environmental Protection, Department of Chemistry, Yancheng Teachers University, Yancheng 224002, People’s Republic of China

## Abstract

The title compound, C_14_H_9_Cl_2_N_3_O_3_, was prepared by the reaction of 3-nitro­benzohydrazide with 2,4-dichloro­benzalde­hyde. The mol­ecule adopts an *E* configuration about the C=N bond. The dihedral angle between the two benzene rings is 4.6 (2)°. In the crystal, the hydrazone mol­ecules are linked through inter­molecular N—H⋯O hydrogen bonds, forming chains along the *c* axis.

## Related literature

For medical applications of hydrazones, see: Ajani *et al.* (2010 ▶); Zhang *et al.* (2010 ▶); Angelusiu *et al.* (2010 ▶). For related structures, see: Huang & Wu (2010 ▶); Khaledi *et al.* (2010 ▶); Zhou & Yang (2010 ▶); Ji & Lu (2010 ▶); Singh & Singh (2010 ▶); Ahmad *et al.* (2010 ▶). For similar compounds that we have reported recently, see: Dai & Mao (2010*a* ▶,*b* ▶).
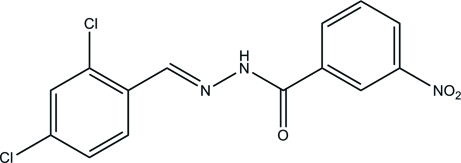


## Experimental

### 

#### Crystal data

C_14_H_9_Cl_2_N_3_O_3_
*M* *_r_* = 338.14
Monoclinic, 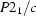

*a* = 12.004 (3) Å
*b* = 14.384 (3) Å
*c* = 8.465 (2) Å
β = 96.302 (2)°
*V* = 1452.8 (6) Å^3^
*Z* = 4
Mo *K*α radiation
μ = 0.46 mm^−1^
*T* = 298 K
0.20 × 0.20 × 0.18 mm

#### Data collection

Bruker SMART CCD area-detector diffractometer
Absorption correction: multi-scan (*SADABS*; Bruker, 2001 ▶) *T*_min_ = 0.913, *T*_max_ = 0.921
11555 measured reflections
3163 independent reflections
2128 reflections with *I* > 2σ(*I*)
*R*_int_ = 0.038

#### Refinement

*R*[*F* ^2^ > 2σ(*F* ^2^)] = 0.043
*wR*(*F* ^2^) = 0.107
*S* = 1.05
3163 reflections
202 parameters
1 restraint
H atoms treated by a mixture of independent and constrained refinement
Δρ_max_ = 0.22 e Å^−3^
Δρ_min_ = −0.20 e Å^−3^

### 

Data collection: *SMART* (Bruker, 2007 ▶); cell refinement: *SAINT* (Bruker, 2007 ▶); data reduction: *SAINT*; program(s) used to solve structure: *SHELXTL* (Sheldrick, 2008 ▶); program(s) used to refine structure: *SHELXTL*; molecular graphics: *SHELXTL*; software used to prepare material for publication: *SHELXTL*.

## Supplementary Material

Crystal structure: contains datablocks global, I. DOI: 10.1107/S1600536810049470/sj5069sup1.cif

Structure factors: contains datablocks I. DOI: 10.1107/S1600536810049470/sj5069Isup2.hkl

Additional supplementary materials:  crystallographic information; 3D view; checkCIF report

## Figures and Tables

**Table 1:** Hydrogen-bond geometry (Å, °)

*D*—H⋯*A*	*D*—H	H⋯*A*	*D*⋯*A*	*D*—H⋯*A*
N2—H2⋯O1^i^	0.89 (1)	2.04 (2)	2.859 (2)	152 (2)

Symmetry code: (i) 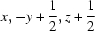.

**Fig. 1.**
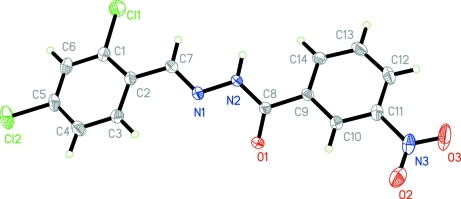
The molecular structure of the title compound showing 30% probability displacement ellipsoids and the atomic numbering.

**Fig. 2.**
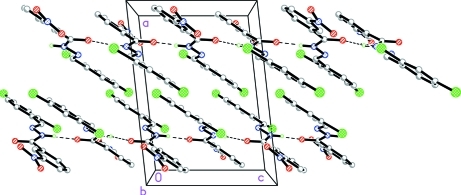
Crystal packing of the title compound, viewed down the b axis. Intermolecular interactions are drawn as dashed lines.

## supplementary crystallographic information

### Comment

In the last few years, medical applications of a number of hydrazone compounds have been received much attention (Ajani *et al.*, 2010; Zhang *et al.*, 2010; Angelusiu *et al.*, 2010). The structures of several hydrazone derivatives have also been determined (Huang & Wu, 2010; Khaledi *et al.*, 2010; Zhou & Yang, 2010; Ji & Lu, 2010; Singh & Singh, 2010; Ahmad *et al.*, 2010). As a continuation of our work on this area (Dai & Mao, 2010*a*,*b*), in this paper, we report the structure of the new derivative *E**N*'-(2,4-dichlorobenzylidene)-3-nitrobenzohydrazide.

In the molecule of the title compound, the dihedral angle between the C1···C6 and C9···C14 benzene rings is 4.6 (2)°. The O2/N3/O3 plane forms a dihedral angle of 8.9 (2)° with the C9···C14 benzene ring. The bond lengths and angles are comparable to those found in the hydrazone compounds cited above. In the crystal structure, the hydrazone molecules are linked through intermolecular N—H···O hydrogen bonds (Table 1), to form one-dimensional chains along the *c* axis, as shown in Fig. 2.

### Experimental

The reaction of 3-nitrobenzohydrazide (0.181 g, 1 mmol) with 2,4-dichlorobenzaldehyde (0.174 g, 1 mmol) in 50 ml methanol at room temperature afforded the title compound. Yellow block-shaped single crystals were formed by slow evaporation of the clear solution in air.

### Refinement

The amino H atom was located in a difference Fourier map and refined with N—H = 0.90 (1) Å, and *U*_iso_ = 0.08 Å^2^. Other H atoms were positioned geometrically (C—H = 0.93 Å) and refined as riding with *U*_iso_(H) = 1.2*U*_eq_(C).

### Crystal data

**Table:**

C_14_H_9_Cl_2_N_3_O_3_	*F*(000) = 688
*M**_r_* = 338.14	*D*_x_ = 1.546 Mg m^−^^3^
Monoclinic, *P*2_1_/*c*	Mo *K*α radiation, λ = 0.71073 Å
Hall symbol: -P 2ybc	Cell parameters from 2237 reflections
*a* = 12.004 (3) Å	θ = 2.3–24.5°
*b* = 14.384 (3) Å	µ = 0.46 mm^−^^1^
*c* = 8.465 (2) Å	*T* = 298 K
β = 96.302 (2)°	Block, yellow
*V* = 1452.8 (6) Å^3^	0.20 × 0.20 × 0.18 mm
*Z* = 4	

### Data collection

**Table:**

Bruker SMART CCD area-detector diffractometer	3163 independent reflections
Radiation source: fine-focus sealed tube	2128 reflections with *I* > 2σ(*I*)
graphite	*R*_int_ = 0.038
ω scans	θ_max_ = 27.0°, θ_min_ = 2.2°
Absorption correction: multi-scan (*SADABS*; Bruker, 2001)	*h* = −15→13
*T*_min_ = 0.913, *T*_max_ = 0.921	*k* = −18→18
11555 measured reflections	*l* = −10→10

### Refinement

**Table:**

Refinement on *F*^2^	Primary atom site location: structure-invariant direct methods
Least-squares matrix: full	Secondary atom site location: difference Fourier map
*R*[*F*^2^ > 2σ(*F*^2^)] = 0.043	Hydrogen site location: inferred from neighbouring sites
*wR*(*F*^2^) = 0.107	H atoms treated by a mixture of independent and constrained refinement
*S* = 1.05	*w* = 1/[σ^2^(*F*_o_^2^) + (0.0467*P*)^2^ + 0.1033*P*] where *P* = (*F*_o_^2^ + 2*F*_c_^2^)/3
3163 reflections	(Δ/σ)_max_ < 0.001
202 parameters	Δρ_max_ = 0.22 e Å^−^^3^
1 restraint	Δρ_min_ = −0.20 e Å^−^^3^

### Special details

**Table:**

Geometry. All esds (except the esd in the dihedral angle between two l.s. planes) are estimated using the full covariance matrix. The cell esds are taken into account individually in the estimation of esds in distances, angles and torsion angles; correlations between esds in cell parameters are only used when they are defined by crystal symmetry. An approximate (isotropic) treatment of cell esds is used for estimating esds involving l.s. planes.
Refinement. Refinement of F^2^ against ALL reflections. The weighted R-factor wR and goodness of fit S are based on F^2^, conventional R-factors R are based on F, with F set to zero for negative F^2^. The threshold expression of F^2^ > 2sigma(F^2^) is used only for calculating R-factors(gt) etc. and is not relevant to the choice of reflections for refinement. R-factors based on F^2^ are statistically about twice as large as those based on F, and R- factors based on ALL data will be even larger.

### Fractional atomic coordinates and isotropic or equivalent isotropic displacement parameters (Å^2^)

**Table:**

	*x*	*y*	*z*	*U*_iso_*/*U*_eq_	
Cl1	0.26406 (6)	−0.09815 (4)	0.11977 (8)	0.0651 (2)	
Cl2	0.49631 (6)	−0.18532 (5)	−0.36654 (9)	0.0760 (3)	
N1	0.28128 (14)	0.18486 (11)	−0.03316 (19)	0.0344 (4)	
N2	0.23633 (14)	0.25093 (11)	0.06051 (18)	0.0342 (4)	
N3	0.12644 (17)	0.66091 (13)	0.0801 (3)	0.0542 (5)	
O1	0.20635 (13)	0.34676 (9)	−0.15308 (16)	0.0454 (4)	
O2	0.19086 (19)	0.67781 (12)	−0.0175 (3)	0.0808 (6)	
O3	0.06980 (18)	0.71959 (12)	0.1368 (3)	0.0922 (7)	
C1	0.33504 (17)	−0.06183 (14)	−0.0373 (3)	0.0385 (5)	
C2	0.34878 (16)	0.03282 (13)	−0.0659 (2)	0.0330 (5)	
C3	0.40982 (18)	0.05744 (14)	−0.1909 (2)	0.0394 (5)	
H3	0.4201	0.1200	−0.2128	0.047*	
C4	0.45528 (18)	−0.00914 (16)	−0.2829 (2)	0.0449 (5)	
H4	0.4973	0.0084	−0.3640	0.054*	
C5	0.43747 (18)	−0.10158 (15)	−0.2525 (3)	0.0446 (6)	
C6	0.37757 (18)	−0.12984 (15)	−0.1318 (3)	0.0451 (6)	
H6	0.3657	−0.1926	−0.1135	0.054*	
C7	0.30075 (17)	0.10478 (13)	0.0282 (2)	0.0353 (5)	
H7	0.2847	0.0923	0.1311	0.042*	
C8	0.20044 (16)	0.33100 (13)	−0.0112 (2)	0.0320 (5)	
C9	0.15054 (16)	0.40225 (13)	0.0894 (2)	0.0296 (4)	
C10	0.16380 (16)	0.49489 (13)	0.0476 (2)	0.0340 (5)	
H10	0.2056	0.5105	−0.0347	0.041*	
C11	0.11400 (18)	0.56310 (14)	0.1300 (2)	0.0386 (5)	
C12	0.05041 (19)	0.54271 (16)	0.2516 (3)	0.0490 (6)	
H12	0.0173	0.5898	0.3054	0.059*	
C13	0.03713 (19)	0.45074 (17)	0.2916 (3)	0.0477 (6)	
H13	−0.0056	0.4356	0.3732	0.057*	
C14	0.08693 (16)	0.38009 (15)	0.2110 (2)	0.0376 (5)	
H14	0.0774	0.3183	0.2388	0.045*	
H2	0.231 (2)	0.2398 (18)	0.1627 (14)	0.080*	

### Atomic displacement parameters (Å^2^)

**Table:**

	*U*^11^	*U*^22^	*U*^33^	*U*^12^	*U*^13^	*U*^23^
Cl1	0.0776 (5)	0.0440 (4)	0.0797 (5)	−0.0040 (3)	0.0359 (4)	0.0085 (3)
Cl2	0.0832 (5)	0.0620 (4)	0.0845 (5)	0.0183 (4)	0.0174 (4)	−0.0351 (4)
N1	0.0418 (10)	0.0307 (9)	0.0318 (9)	0.0035 (8)	0.0095 (8)	−0.0038 (7)
N2	0.0493 (11)	0.0294 (9)	0.0259 (8)	0.0063 (8)	0.0126 (8)	0.0000 (7)
N3	0.0544 (14)	0.0335 (11)	0.0733 (15)	0.0013 (10)	0.0004 (11)	−0.0153 (10)
O1	0.0735 (11)	0.0371 (8)	0.0285 (8)	0.0118 (7)	0.0187 (7)	0.0037 (6)
O2	0.1069 (17)	0.0392 (10)	0.1022 (16)	−0.0029 (10)	0.0370 (14)	0.0029 (10)
O3	0.0933 (15)	0.0387 (10)	0.151 (2)	0.0116 (10)	0.0411 (14)	−0.0234 (12)
C1	0.0363 (12)	0.0334 (11)	0.0455 (12)	0.0019 (9)	0.0034 (10)	0.0002 (9)
C2	0.0334 (11)	0.0326 (11)	0.0324 (11)	0.0044 (9)	0.0011 (9)	−0.0038 (8)
C3	0.0489 (14)	0.0332 (11)	0.0365 (12)	0.0037 (10)	0.0059 (10)	−0.0011 (9)
C4	0.0486 (14)	0.0508 (14)	0.0365 (12)	0.0040 (11)	0.0098 (10)	−0.0078 (10)
C5	0.0425 (13)	0.0420 (13)	0.0484 (13)	0.0102 (10)	0.0013 (11)	−0.0168 (10)
C6	0.0474 (14)	0.0297 (11)	0.0568 (14)	0.0037 (10)	−0.0002 (12)	−0.0072 (10)
C7	0.0415 (13)	0.0348 (11)	0.0301 (11)	0.0036 (9)	0.0068 (9)	−0.0008 (8)
C8	0.0382 (12)	0.0286 (10)	0.0299 (11)	−0.0011 (9)	0.0066 (9)	−0.0021 (8)
C9	0.0300 (11)	0.0327 (11)	0.0258 (10)	0.0025 (8)	0.0024 (8)	−0.0012 (8)
C10	0.0338 (11)	0.0334 (11)	0.0351 (11)	−0.0003 (9)	0.0048 (9)	−0.0041 (8)
C11	0.0376 (12)	0.0341 (11)	0.0426 (12)	0.0050 (9)	−0.0017 (10)	−0.0076 (9)
C12	0.0478 (14)	0.0534 (15)	0.0460 (13)	0.0155 (11)	0.0054 (11)	−0.0160 (11)
C13	0.0452 (14)	0.0649 (16)	0.0355 (12)	0.0095 (12)	0.0155 (10)	−0.0011 (11)
C14	0.0391 (12)	0.0430 (12)	0.0315 (11)	0.0030 (10)	0.0070 (9)	0.0031 (9)

### Geometric parameters (Å, °)

**Table:**

Cl1—C1	1.736 (2)	C4—C5	1.376 (3)
Cl2—C5	1.741 (2)	C4—H4	0.9300
N1—C7	1.275 (2)	C5—C6	1.374 (3)
N1—N2	1.384 (2)	C6—H6	0.9300
N2—C8	1.350 (2)	C7—H7	0.9300
N2—H2	0.889 (10)	C8—C9	1.499 (3)
N3—O3	1.215 (2)	C9—C14	1.384 (3)
N3—O2	1.216 (3)	C9—C10	1.392 (3)
N3—C11	1.481 (3)	C10—C11	1.378 (3)
O1—C8	1.232 (2)	C10—H10	0.9300
C1—C6	1.395 (3)	C11—C12	1.379 (3)
C1—C2	1.396 (3)	C12—C13	1.379 (3)
C2—C3	1.397 (3)	C12—H12	0.9300
C2—C7	1.463 (3)	C13—C14	1.395 (3)
C3—C4	1.383 (3)	C13—H13	0.9300
C3—H3	0.9300	C14—H14	0.9300
			
C7—N1—N2	116.89 (16)	N1—C7—C2	118.86 (18)
C8—N2—N1	116.96 (15)	N1—C7—H7	120.6
C8—N2—H2	122.3 (17)	C2—C7—H7	120.6
N1—N2—H2	120.7 (17)	O1—C8—N2	123.05 (17)
O3—N3—O2	123.7 (2)	O1—C8—C9	119.84 (17)
O3—N3—C11	117.9 (2)	N2—C8—C9	117.11 (17)
O2—N3—C11	118.37 (19)	C14—C9—C10	119.82 (18)
C6—C1—C2	121.8 (2)	C14—C9—C8	123.54 (17)
C6—C1—Cl1	117.98 (16)	C10—C9—C8	116.48 (17)
C2—C1—Cl1	120.26 (16)	C11—C10—C9	118.99 (19)
C1—C2—C3	117.44 (18)	C11—C10—H10	120.5
C1—C2—C7	122.28 (19)	C9—C10—H10	120.5
C3—C2—C7	120.28 (18)	C10—C11—C12	122.2 (2)
C4—C3—C2	121.5 (2)	C10—C11—N3	118.0 (2)
C4—C3—H3	119.2	C12—C11—N3	119.8 (2)
C2—C3—H3	119.2	C11—C12—C13	118.4 (2)
C5—C4—C3	119.0 (2)	C11—C12—H12	120.8
C5—C4—H4	120.5	C13—C12—H12	120.8
C3—C4—H4	120.5	C12—C13—C14	120.8 (2)
C6—C5—C4	122.0 (2)	C12—C13—H13	119.6
C6—C5—Cl2	119.02 (17)	C14—C13—H13	119.6
C4—C5—Cl2	118.97 (19)	C9—C14—C13	119.8 (2)
C5—C6—C1	118.3 (2)	C9—C14—H14	120.1
C5—C6—H6	120.9	C13—C14—H14	120.1
C1—C6—H6	120.9		

### Hydrogen-bond geometry (Å, °)

**Table:**

*D*—H···*A*	*D*—H	H···*A*	*D*···*A*	*D*—H···*A*
N2—H2···O1^i^	0.89 (1)	2.04 (2)	2.859 (2)	152 (2)

Symmetry codes: (i) *x*, −*y*+1/2, *z*+1/2.
